# Abnormally Methylated *FMR1* in Absence of a Detectable Full Mutation in a U.S.A Patient Cohort Referred for Fragile X Testing

**DOI:** 10.1038/s41598-019-51618-7

**Published:** 2019-10-25

**Authors:** Charles H. Hensel, Rena J. Vanzo, Megan M. Martin, Ling Ling, Solange M. Aliaga, Minh Bui, David I. Francis, Hope Twede, Michael H. Field, Jonathon W. Morison, David J. Amor, David E. Godler

**Affiliations:** 1 Lineagen, Inc., Salt Lake City, UT United States of America; 2 Diagnosis and Development, Murdoch Children’s Research Institute, Royal Children’s Hospital, Melbourne, VIC Australia; 3 Centre for Epidemiology and Biostatistics, Melbourne School of Population and Global Health, University of Melbourne, Melbourne, VIC Australia; 4 Victorian Clinical Genetics Services and Murdoch Children’s Research Institute, Royal Children’s Hospital, Melbourne, VIC Australia; 5 Genetics of Learning Disability Service (GOLD service), Hunter Genetics, Newcastle, NSW Australia; 6 Business Development and Legal Office, Murdoch Children’s Research Institute, Royal Children’s Hospital, Melbourne, VIC, Australia; 7 Faculty of Medicine, Dentistry and Health Sciences, Department of Paediatrics, University of Melbourne, Parkville, VIC Australia; 8 Neurodisability and Rehabilitation Research Group, Murdoch Children’s Research Institute, Royal Children’s Hospital, Melbourne, VIC Australia

**Keywords:** Diagnostic markers, Genetics research

## Abstract

In 2016, Methylation-Specific Quantitative Melt Analysis (MS-QMA) on 3,340 male probands increased diagnostic yield from 1.60% to 1.84% for fragile X syndrome (FXS) using a pooling approach. In this study probands from Lineagen (UT, U.S.A.) of both sexes were screened using MS-QMA without sample pooling. The cohorts included: (i) 279 probands with no FXS full mutation (FM: CGG > 200) detected by AmplideX CGG sizing; (ii) 374 negative and 47 positive controls. MS-QMA sensitivity and specificity in controls approached 100% for both sexes. For male probands with no FM detected by standard testing (n = 189), MS-QMA identified abnormal DNA methylation (mDNA) in 4% normal size (NS: < 44 CGGs), 6% grey zone (CGG 45–54) and 12% premutation (CGG 54–199) alleles. The abnormal mDNA was confirmed by AmplideX methylation sensitive (m)PCR and EpiTYPER tests. In contrast, no abnormal mDNA was detected in 89 males with NS alleles from the general population. For females, 11% of 43 probands with NS alleles by the AmplideX sizing assay had abnormal mDNA by MS-QMA, with FM / NS mosaicism confirmed by AmplideX mPCR. *FMR1* MS-QMA analysis can cost-effectively screen probands of both sexes for methylation and FM mosaicism that may be missed by standard testing.

## Introduction

Intellectual disability (ID) and/or autism spectrum disorders (ASD) are identified in ~1.7% of births globally^1^, representing a significant burden to families and resulting in a major cost to the economy^2^. Although recent advances in Next Generation Sequencing (NGS) technologies have markedly improved diagnostic rates, a specific cause remains elusive in >50% of all children with ID/ASD^1^. Many mutations in known ID/ASD genes may go unnoticed because only a small proportion of cells in the body harbour them. This effect, known as low level mosaicism (LLM), may be present in the brain without being readily detectable in the tissues commonly sampled for genetic analysis (e.g. blood and saliva). These changes can include: (i) DNA sequence or allele copy number alterations; and/or (ii) DNA methylation that affects gene regulation. While chromosomal microarray analysis (CMA) and Whole Exome Sequencing (WES) are commonly utilized tests for developmental delay (DD) referrals, they are limited by inability to detect changes in: (i) clinically significant LLM, present in as little as 5% of the affected tissue^3^; and (ii) changes in large repetitive DNA sequences. Thus, the *FMR1* CGG repeat expansion PCR test is usually performed separately from CMA and WES for all DD referrals.

When the *FMR1* CGG sequence on the X chromosome is ≥200 CGG repeats, known as full mutation (FM), it causes silencing of *FMR1* through DNA methylation^4^. Loss of *FMR1* protein (FMRP) results in fragile X syndrome (FXS) - a common single gene cause of ID. FXS is found in ~1 in 4000 in the general population^5^. FMRP regulates synaptic function^5^, and outside of FXS has been implicated in key pathways associated with neurological disorders^6,7^.

*FMR1* alleles in the 55–199 CGG range are classified as a premutation (PM), and can cause Fragile X-Associated Tremor/Ataxia Syndrome (FXTAS) and Fragile X-Associated Primary Ovarian Insufficiency (FXPOI)^8^. Recent studies have reported PM alleles to be present in approximately 1 in 500 males, and between 1 in 120 and 1 in 300 females in the general population^8^. Importantly, PM alleles in females have been associated with an increased risk of having a child with a FM affected with FXS. In contrast, CGG repeats in the 45–54 range classified as gray zone (GZ) or intermediate alleles, have not been reported to expand to FM in a single generation, nor have they been definitively linked to any phenotype. However, GZ alleles are more common, found in up to 1 in 125 males and 1 in 40 females in the general population^8^. Normal size alleles are classified as having 44 CGG repeats or fewer, with the 31 CGG repeat size being the most common allele^8^.

Current diagnostic testing for FXS usually involves a stepwise process of first determining CGG repeat size, usually by AmplideX PCR or another PCR based method, followed by assaying *FMR1* promoter methylation status using AmplideX mPCR with or without Southern blot based analysis targeting the *FMR1* CpG island (with the analytical sensitivity at 5–20%). In 2014, a novel cost-effective methodology called Methylation Specific Quantitative Melt Analysis (MS-QMA) was shown to detect the presence of abnormally methylated alleles present in as few as 1% of cells. This methodology significantly increased the diagnostic yield for FXS in pooled male probands that were negative by standard testing^9,10^.

MS-QMA examines a different region of the *FMR1* promoter, called the Fragile X Related epigenetic element 2 (FREE2), located at the *FMR1* exon1/intron 1 boundary (reviewed in^11^). This study expands on the previous studies^9^ by using MS-QMA as a first line test for probands without sample pooling, in both sexes, in an independent cohort of probands from the U.S.A. MS-QMA testing was performed on GZ, PM as well as normal *FMR1* allele size probands, with the results for the first time compared to methylation analysis using long-range AmplideX methylation specific (m)PCR, as well as CMA and EpiTYPER system methylation, which were not evaluated in the previous studies^9,10,12^.

## Results

### Abnormal MS-QMA methylation in male DD cohort

**Figure 1:**
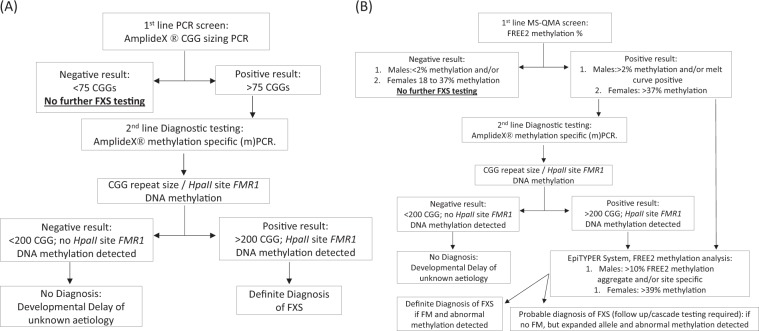
The two FXS testing workflows. (**A**) Standard screening and testing workflow with CGG based 1^st^ line screening test for the U.S.A. DD cohort utilized in this study; (**B**) Study workflow with FREE2 methylation assessed using MS-QMA as the 1^st^ line screening test.

**Figure 2:**
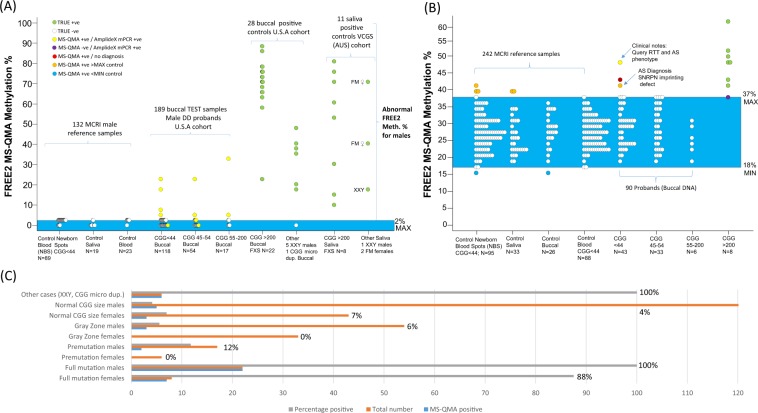
MS-QMA testing of: (**A**) U.S.A. cohort males with: DD negative by standard FXS testing (n = 189), FM and confirmed FXS diagnosis (n = 22), males with Klinefelter syndrome (n = 6), one CGG microduplication with normal karyotype. Australian cohort included male reference samples with normal CGG size including typically developing (IQ > 70) controls (n = 42) and newborn blood spots from the general population (n = 89), as well as 2 FM female and 8 FM male positive controls from and one Klinefelter syndrome patient samples. *Note: “*+*ve”* = *positive*; *“*−*ve”* = *negative*. Confirmatory testing was performed using AmplideX mPCR, and MALDI-TOF MS EpiTYPER system F*MR1* methylation analysis. Male control reference range indicated in light blue (mean ± 2 standard deviations): 2 ± 0.01%. Below 2% methylation positive calls were made from raw data assessment of MS-QMA derivative curves. (**B**) U.S.A. cohort females included: DD negative by standard FXS testing (n = 90), FM and confirmed FXS diagnosis (n = 8), males with Klinefelter syndrome. Australian cohort included female reference samples with normal CGG size including typically developing controls (IQ > 70; n = 33) and newborn blood spots from the general population (n = 95). (**C**) Percentage and the number of MS-QMA positive, as well as the total number of cases tested for the U.S.A. cohort in each allelic group for males and females. *Note:* DD = developmental delay referral; RTT = Rett syndrome; AS = Angelman syndrome; true positives: FXS males and females confirmed to have FM alleles by both AmplideX CGG screening assay and mPCR. Female control reference range indicated in light blue (mean ± 2 standard deviations): 27 ± 9%, or between 18% and 37% representing the normal amount of FREE2 methylation attributed to X chromosome inactivation, consistent with the range reported in previous studies^12^.

Post unblinding, all 21 typical FXS FM males and 6 Klinefelter syndrome males from Lineagen (UT, U.S.A.) positive by AmplideX CGG sizing (Fig. 1A), as well as, 9 Victorian Clinical Genetics Services (VCGS) male positive controls (8 FM and one Klinefelter syndrome males), were also positive by MS-QMA, with FREE2 methylation above 2% (the maximum value from blood DNA of 23 typically developing males (FSIQ > 70) with normal size alleles by standard testing) (Fig. 2A). As expected, a male with a CGG micro-duplication from Lineagen (UT, U.S.A.) had FREE2 methylation within the normal methylation range.

**Figure 3:**
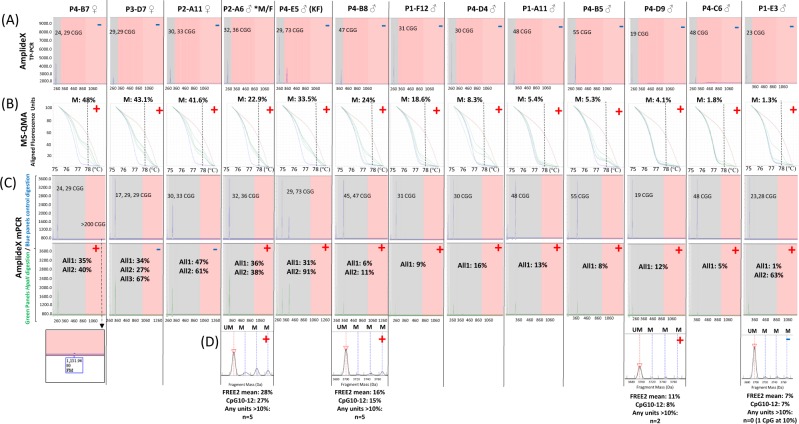
Raw profiles, CGG sizes and methylation levels for thirteen cases with developmental delay identified to have abnormal methylation using MS-QMA, that were negative by 1^st^ line testing using AmplideX triplet-repeat primed PCR (TP-PCR) commercial assay. For each panel + indicates a positive result; − indicates a negative result. AmplideX mPCR a positive result was defined as presence of: (i) abnormal methylation with or without FM in males (typically developing males have 0% methylation); (ii) presence of a FM in females. M = methylated peak. Each column indicates a different technique: (**A**) AmplideX TP-PCR; (**B**) MS-QMA; (**C**) AmplideX mPCR, with upper panels with blue traces representing control digestion and lower panels with green traces representing peaks from *HpaII* digestion reactions. These blue and green traces were used to determine average methylation across two *HpaII* sites, 5′ and 3′ of the CGG expansion using Gene Mapper software version 5.0 (Life Technologies, Foster City, CA), as per manufacturer’s instructions (Asuragen, Austin, Texas, USA). The raw profiles that include regions for visual assessment for the completeness of *HpaII* digestion controls have been included in the Fig. S1. (**D**) EpiTYPER system. Each column represents a different sample, with sample ID included in row 1. P4-B7; P3-D7 and P2-A11 were female referrals as indicated by ♀ in row 1, with abnormal MS-QMA signatures. The other cases were male referrals indicated by ♂ in row 1. Note: M methylation = mean *HpaII* methylation across detected alleles; *M/F = male that was biologically born as female; KF = Klinefelter syndrome. The vertical line superimposed onto MS-QMA profiles indicates the melt temperature of 78 °C used to differentiate between 100% and 0% methylated alleles, as previously described^9^; brown and blue melt curves represent FM and normal CGG size (<44 repeats) control samples, 100% and 0% methylated respectively. While green melt curves represent high resolution melt profiles used to determine % methylation by MS-QMA for the test samples, with 4 melt curve per sample representing two separate bisulfite conversion technical replicates, with each conversion having two melt curves from two serial dilutions, used to calculate % methylation by the Q-MAX software, as previously described^10^. All1 and All2 represent smaller and larger size alleles respectively. For sample P4-B7 of a female with developmental delay an unmethylated FM allele was detected by AmplideX mPCR but not by standard AmplideX TP-PCR. For the EpiTYPER system red triangles and red dotted lines represent mean peak height of the unmethylated fragment for FREE2 CpG10, 11 and 12 with positive calles based on methylation data presented for: (I) mean values across the 12 CpG sites; (II) CpG10–12 unit (previously shown to have the most significant correlation with intellectual functioning of all sites examined)^17^; (III) the number of CpG sites that had methylation above 10% maximum value of the control population examined in previous studies^17^. Note: pink background for panels in (**A**) represents peaks for alleles of PM CGG size and above (>54 CGGs); while for panels in (**C**) represent peaks for alleles of FM CGG size and above (>199 CGGs). Gray background represents peaks for alleles of <55 and <200 CGGs, for panels in (**A**) and (**C**) respectively.

MS-QMA also identified ten male samples with abnormal MS-QMA signatures that were negative using the AmplideX 1^st^ line CGG sizing screen (Table 1): five (4%) with normal CGG repeats, three (6%) with the GZ alleles and 2 (12%) with PM alleles (Figs 2A,C and 3). Clinical notes for these 10 male probands indicated that the most common reasons for referral were: DD (2/10), ID (3/10), and ASD (2/10) (Table 1). One male with an abnormally methylated PM allele of 73 CGGs (ID: P4-E5), also had a diagnosis of Klinefelter syndrome by CMA (Table 1). Another male (ID: P2-A6) from subsequent follow-up was found to have been biologically born as a female, explaining the unexpected MS-QMA result approaching 23% methylation.

**Table 1:** Characteristics of the thirteen cases with developmental delay (DD) and abnormal FREE2 MS-QMA methylation results, that were negative by AmplideX TP-PCR as part of standard fragile X 1^st^ line PCR testing.

ID:	Sex	Age (years)	Reason for referral/Clinical notes	Probands CGG size: AmplideX TP-PCR 1^st^ line test	Family history (if available)	CMA result	Another diagnosis (if available)	Confirmation status of the abnormal MS-QMA result (Y/N)
P4-B7	F	2	DD, ASD, query Rett/Angelman syndrome phenotype.	24, 29	N/A	Normal		Y [AmplideX mPCR; FM detected]
P3-D7	F	0.83	DD	29, 29	N/A	arr[hg19]15q11.2q13.1(22,770,421–28,526,410)x1	Angelman syndrome	N
P2-A11	F	16	ID, epilepsy	30, 33	N/A	Normal	N/A	N
P2-A6*	M/F*	24	Spina bifida; medical notes indicate born biologically female	32, 36	N/A	Normal		Y [AmplideX mPCR; EpiTYPER]
P4-E5	M	14	Tall stature, learning disability, connective tissue disorder	29, 73	N/A	arr[hg19](1–22,X)x2,Yx1	Klinefelter syndrome	Y [AmplideX mPCR]
P4-B8	M	8	ASD	47	N/A	arr[hg19] 2q13(110,873,834–110,980,919)x1	Variant of uncertain significance	Y [AmplideX mPCR; EpiTYPER]
P1-F12	M	10	ADHD	31	N/A	Normal		Y [AmplideX mPCR]
P4-D4	M	7	ID	30		Normal		Y [AmplideX mPCR]
P1-A11	M	3	DD, ASD, epilepsy	48	carrier fragile X mother, DD FHx.	arr[hg19]13q32.3(101,173,173–101,270,648)x1	Variant of uncertain significance	Y [AmplideX mPCR]
P4-B5	M	2	DD	55	N/A	Normal		Y [AmplideX mPCR]
P4-D9	M	16	DD, epilepsy	19	N/A	arr[hg19]1q44(243,804,016–244,844,380)x1	1q44 deletion	Y [AmplideX mPCR; EpiTYPER]
P4-C6	M	11	ID	48	N/A	Normal		Y [AmplideX mPCR]
P1-E3	M	7	ID, ASD	23	N/A	Normal		Y [AmplideX mPCR]

Note: Abnormal MS-QMA results were considered confirmed if also positive by AmplideX mPCR and/or FREE2 methylaiton EpiTYPER analysis. Sex (on the referral) column: M = male; F = female. ID = intellectual disability; DD = developmental delay, ADHD = attention deficit hyperactivity disorder; ASD = autism spectrum disorder; N/A = not available; *M/F = male that was biologically born as female.

From the 10 additional MS-QMA positive male samples, nine had FREE2 methylation ranging between 2% and 30%, while one case (ID: P1-E3) with normal CGG size had methylation below 2%. This patient had an abnormal melt curve, with higher fluorescence from visual inspection at 78 *°C* as compared to normal size control, suggesting the presence of LLM (Fig. 2). Importantly, all 10 additional MS-QMA positive cases also had abnormal *FMR1* methylation by AmplideX mPCR, with 4 of these also showing abnormal FREE2 methylation using the EpiTYPER system (Fig. 3A,C). One male (ID: P1-A11) with an intermediate size allele and MS-QMA methylation close to 5% had a mother who was reported to carry a non-normal allele as part of carrier screening. Another male (P1-E3) was found to have a second allele with 28 CGG, 63% methylated by AmpideX mPCR, that was not detected by the AmplideX CGG screening test (Figs 3 and 1S).

For the 89 male newborn blood spots with normal size alleles from the general population, none of the values were >2% maximum methylation value, and none had an abnormal melt curve, with higher fluorescence from visual inspection at 78 *°C* as compared to the whole blood DNA from control males with full scale IQ (FSIQ) > 70, assessed as part of our previous studies^13–15^.

### Abnormal MS-QMA methylation in the female DD cohort

Post un-blinding, 7 typical FXS FM female probands from Lineagen (UT, U.S.A.) positive by AmplideX CGG sizing (Fig. 2B), as well as, 2 VCGS FM female positive controls (Fig. 2A), were also positive by MS-QMA, with FREE2 methylation above 37% (the maximum value of the female normal methylation range in blood of typically developing female controls with full scale IQ (FSIQ) > 70) (Fig. 2A). The 37% methylation cut-off also equated to 2 standard deviations above the mean methylation value for female newborn bloods spots, as well as buccal epithelial cell and saliva DNA extracted from typically developing female controls (Fig. 2B).

MS-QMA also identified three female probands with abnormal MS-QMA signatures out of the 43 tested that had only normal size alleles detected by the AmplideX 1^st^ line CGG sizing screen (Table 1 and Fig. 2B). This equates to 7% of female probands with *FMR1* allele sizes in the normal CGG range by the standard AmplideX CGG screening test. Follow-up testing using AmplideX mPCR identified a FM allele in one of the three females. This female (ID: P4-B7) had the highest MS-QMA methylation of 48% (Figs 2B and 3), as well as the smallest normal size allele of 24 CGG repeats from the three additional cases identified by MS-QMA. Interestingly, this female (ID: P4-B7) did not have a classical FXS presentation and was referred for testing with Rett/Angelman syndrome-like features (Table 1). Furthermore, another female with an abnormal MS-QMA result of 43% (ID: P3-D7) had a confirmed molecular diagnosis of Angelman syndrome due to 15q11-13 deletion (Table 1), but also had the 3^rd^ allele of 17 CGG repeats that was 67% methylated, detected by AmplideX mPCR, that was not detected by the AmplideX CGG screening test (Figs 3 and 1S).

In 96 female NBS from the general population with normal size alleles by standard testing, 3 samples (3%) had MS-QMA methylation values slightly above the methylation maximum value found in blood of typically developing female controls, assessed as part of previous studies^16–18^.

### Sensitivity and specificity for FM alleles: MS-QMA versus AmplideX CGG screening assay

For probands tested from the Lineagen cohort, all 21 FXS FM males, and 7 out of 8 FM females with FM identified by AmplideX CGG sizing screening test were also positive by MS-QMA. The one FM female that was called as negative by MS-QMA (following data un-blinding) had 37% methylation, which is the maximum value of the control range (the positive threshold cut-off). This equates to 97% sensitivity, using 37% as the threshold to differentiate between MS-QMA positive and negative cases. Additionally, no false positives were found in 88 typically developing control females and 23 males (FSIQ > 70), resulting in a specificity of 100%. Alternatively, if MS-QMA methylation ≥ 37%, were used, MS-QMA sensitivity and specificity in this study would be 99.1% and 100%, respectively. In this case, only one out of 88 typically developing control females (FSIQ > 70) had methylation of 37%, and no control males had methylation above 2% positive threshold for males, which would equate to 0.9% false positive rate for samples referred for second line testing.

Importantly, MS-QMA identified one FXS female that was missed by the AmplideX CGG sizing assay, but was confirmed to have a FM size allele mosaicism by AmplideX mPCR second line testing following the positive MS-QMA result. Thus, for the Lineagen cohort, the AmplideX CGG sizing assay detected all 21 FXS FM males and 8 out of 9 FM females with FM identified by combination of MS-QMA and AmplideX mPCR. This equates to 97% sensitivity for the AmplideX CGG screening test, if used as a sole 1^st^ line test.

### Relationships between MS-QMA and AmplideX mPCR methylation percentage

MS-QMA examines mean methylation of all alleles, but in 6 times as many CpG sites (12 CpG sites at the exon 1/intron 1 boundary) as AmplideX mPCR. Furthermore, the presence of mosaic alleles that vary in CGG size does not impact performance of the MS-QMA assay because the target amplicon is located downstream of the CGG expansion (Fig. 3A).

**Figure 4:**
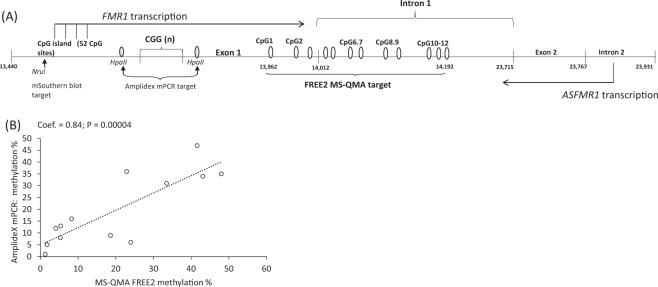
Relationships between FREE2 methylation analysed using MS-QMA and methylation of two *HpaII* sites examined by AmplideX mPCR in thirteen cases identified to have abnormal methylation using MS-QMA. (**A**) Organization of the *FMR1* 5′ region including the CGG expansion (sequence numbering from GenBank L29074 L38501) in relation to *FMR1* and *ASFMR1* transcription start sites, Fragile X Related Epigenetic Element 2 (FREE2) targeted by MS-QMA, the *FMR1* CpG island and methylation sensitive restriction sites and corresponding fragments using routine fragile X by Southern blot testing, two *HpaII* sites targeted by AmplideX methylation PCR. Relationships between FREE2 methylation % (encompassing 12 CpG sites) analysed using MS-QMA with methylation percentage of two *HpaII* methylation sensitive sites targeted by AmplideX mPCR, with (**B**) methylation % of the smallest allele.

For the 13 ‘additional’ methylation and CGG size mosaic cases identified in this study that were negative by the AmplideX CGG screening test, the level of methylation of the smaller alleles by AmplideX mPCR (most cases with a single allele) was significantly correlated with the % methylation detected by MS-QMA (Fig. 4). However, the relationships between MS-QMA output and the AmplideX mPCR results for larger alleles and MS-QMA, were not examined in this study, as the sample size of only 7 cases with two or more alleles was too small for meaningful statistical analyses. Moreover, because the reported resolution of the AmplideX assay is +/−1 CGG repeats some of the additional peaks called as positive within 2 CGG repeat range (for example P4-E5) (Fig. 1S) may represent stutter products from the same allele.

It is important to note that the mPCR results used in this study, were from the AmplideX mPCR assay performed in diagnostic settings at Lineagen, USA, utilizing a commercial kit, with all quality control procedures performed as per manufacturer’s instructions (Asuragen, Inc., Austin, TX, U.S.A.). Specifically, while through visual inspection some samples including P1-E3 (Fig. 1S) appeared to have mPCR peaks for the digestion controls lower than for other samples, expected digestion control peaks for all samples analysed were present, showing >90% digestion, and by this satisfying all manufacturer’s quality control requirements for mPCR analysis. Moreover, methylation of cases analysed by AmplideX mPCR were highly concordant with methylation of the FREE2 region analysed using MS-QMA, including methylation values of <10% (Fig. 4). Because MS-QMA does not employ methylation sensitive restriction enzyme digestion, the close correlation with AmplideX mPCR suggests that there were no major issues associated with digestion controls, for any of the samples that would impact confidence of methylation calculation using data from AmplideX mPCR, even below 10%.

## Discussion

This study shows that analysis of *FMR1* using MS-QMA can effectively screen for the presence of abnormal *FMR1* methylation in both male and female probands with ID and/or ASD referred for FXS testing. While both MS-QMA and the AmplideX CGG sizing screen had diagnostic sensitivity and specificity for FM alleles approaching 100%, both tests also missed one mosaic FXS female (not the same one), with a FM allele confirmed by second line testing. However, in contrast to the AmplideX CGG sizing assay, by altering the positive threshold from 37% to be greater or equal to 37% for MS-QMA, this issue was addressed, increasing the sensitivity to 100% in the tested cohort (at a modest cost of decreasing specificity by <1%).

This study also shows that abnormal *FMR1* methylation determined by MS-QMA in male and female probands is not uncommon even in absence of a FM allele detectable by standard testing. In females, 7% of the 43 female probands negative by the AmplideX CGG screening assay had abnormal methylation signatures by MS-QMA. To gain further support of the clinical significance of these findings, future studies will need to examine if this represents significant enrichment of abnormal *FMR1* methylation in probands in absence of a detectable FM for both sexes when compared to larger cohorts of control newborn blood spots from the general population.

In female probands, two of the three MS-QMA positive female probands with abnormal methylation signatures did not have a FM expansion detected by second line AmplideX mPCR testing. One of these females (P3-D7) had a single peak of 29 CGG detected by standard AmplideX PCR. In contrast AmplideX mPCR provided two methylation output values of 34% and 27%, with two alleles of equal height at ~29 CGGs, and one smaller allele at 17 CGGs that was 67% methylated. This discordance may be due to the mPCR having a better resolution, with the smaller alleles (with fluorescence at 72 units) more clearly visible in the raw data (Sup. Fig. 1), potentially due to the capillary electrophoresis conditions being different for the analysis of the 2 reaction products. Another explanation may be that there is a fourth additional expanded allele (potentially in the FM range) too large to be amplified. This is consistent with the observed skewing such that the smaller 29 and 17 CGG alleles were predominantly found on the active X (at ~30% methylation rather than expected 50%). It is also consistent with the MS-QMA result showing ‘over-methylation’ with the methylation % overlapping with the range in females confirmed to have a hypermethylated FM.

Another female (P4-B7) had atypical presentation, initially referred for testing due to a phenotype reported to be reminiscent of Angelman or Rett syndomes. This female had a FM allele missed by the AmplideX CGG screening assay, and was subsequently identified by second line AmplideX mPCR following observation of the MS-QMA positive result.

In males, 10 additional MS-QMA positive cases were identified in the 189 probands tested. For these cases, when MS-QMA results were stratified based on the allele class, abnormal methylation was found in 4% of patients with a normal size allele, 6% with a GZ allele, and 12% with a PM allele. One potential explanation is that methylated FM alleles in the male probands retracted somatically to smaller size alleles that remained methylated and non-functional. This postulated somatic instability is consistent with: (i) AmplideX mPCR results for two other probands identified (ID: P4-B8 and P2-A6) that had mosaicism (multiple alleles) in the normal and GZ range with methylation 11–100%, and (ii) normal X chromosome copy number by CMA. Importantly, in all additional male probands abnormal methylation of the *FMR1* promoter was confirmed by AmplideX mPCR and the EpiTYPER system, despite the absence of a detectable FM allele. In contrast, none of the 89 general population NBS male samples with normal allele sizes were positive by MS-QMA.

These findings are consistent with another study from two independent cohorts tested using MS-QMA^9^. Abnormal *FMR1* methylation was detected in pooled male DD samples (6 DNA sample aliquots per test). The study identified 18 cases with abnormal *FMR1* methylation, with 8 confirmed by follow up testing of different tissues samples from the probands to have CGG FM expansions and/or their mothers to have PM alleles. This increased the diagnostic yield from 1.60% to 1.84% in 3,384 DD referrals tested, equating to a 15% increase in the total FXS diagnostic yield.

The remaining 10 cases had reproducible abnormal *FMR1* methylation LLM by MS-QMA, but could not be considered FXS due to lack of a detectable FM CGG expansion. Of note, one male from these 10 cases identified in the previous study^9^, had mosaic normal size (29 CGGs) and a PM size (110 CGGs) alleles with abnormal methylation confirmed by multiple methods. This male, mosaic for PM and normal CGG size alleles, had severe intellectual disability (FSIQ 46) but absence of a detectable FM^9^. Similarly, in this study abnormally methylated GZ and PM alleles were detected in males, suggesting that somatic retraction of a FM allele may result in smaller alleles (<200 CGGs in size) that maintain methylation status following retraction. Another explanation is that low level methylation detected by MS-QMA in some of the male probands may represent mosaicism for Klinefelter syndrome. This could include up to 3 males (P4-D9; P4-C6 and P1-E3) of the 10 identified with level close to, or below the level of detection by CMA at ~5% methylation by MS-QMA and AmplideX mPCR.

Future studies should utilise *FMR1* mRNA and FMRP analyses to explore if these smaller alleles that remain hypermethylated have compromised function. Unfortunately, these analyses could not be performed as part of this study as only DNA samples were available from cases referred for fragile X and CMA testing. If compromised *FMR1* function is confirmed in future studies, abnormal methylation in the absence of a detectable FM could be an important novel mechanism leading to FXS. This is in line with the previous literature, where less common intragenic *FMR1* disease-causing variants were also associated with loss of *FMR1* function, leading to FXS in absence of a detectable FM^19^.

Interestingly, for the 13 additional methylation and CGG size mosaic cases identified in this study (where FM alleles were not detected by the AmplideX CGG screening test) methylation % of the smaller size alleles detected by AmplideX mPCR was significantly correlated with MS-QMA methylation %.

In summary, since the early detection and management of FXS is critical for patient care and for providing accurate reproductive advice to the women who carry PM alleles, critical evaluation of current testing strategies should be done to ensure optimal sensitivity. MS-QMA methylation analysis may be considered as an cost-effective alternative that may improve the sensitivity of the current testing paradigm in proband males and females, as commercial mPCR tests are too expensive and of lower throughput to be used for 1^st^ line screening. Future studies should investigate the clinical utility of MS-QMA in the additional cases with abnormal methylation (identified in this and previously studies) that had no FM detected by standard testing^9^. A potential limitation of this study, however, is that cascade testing of family members of most probands identified to have abnormal methylation signatures in this study, has not been performed. Another limitation is that multiple tissue types were not tested, where FM alleles may have been detected, as previously described^9^. However, despite these limitations, and a relatively small sample size in this study, the combined MS-QMA data in this and previous studies^9^ for over 3,500 probands, suggests that commonly utilized testing strategies of screening for FM CGG trinucleotide repeat may miss a substantial number of patients of both sexes who are symptomatic with FXS, and have mothers at risk of having future affected pregnancies.

## Methods

### Participant samples

This study assessed performance of MS-QMA on the U.S.A. cohort comprised of 218 male and 98 female buccal DNA samples collected from probands with developmental delay, between 5 days after birth to 51 years of age (with 97 of cases <18 years of age), referred for fragile X and CMA testing between 2014 and 2017 to Lineagen (UT, U.S.A.). These included 189 male and 90 female probands negative for presence of a FM allele by standard fragile X testing randomly selected from cases referred for testing at Lineagen (UT, U.S.A.). over a 2-week period, as well as 29 male (22 FM; 6 sex chromosome aneuploidy; 1 CGG micro-duplication) and 8 female (FM only) samples with abnormal *FMR1* results confirmed by standard FXS and/or CMA testing at Lineagen (UT, U.S.A.). These samples were clinically evaluated per the standard FXS testing protocol, with the AmplideX CGG sizing assay as the 1^st^ line screening test (Fig. 1A).

The AmplideX results were blinded prior to MS-QMA analysis performed at the Murdoch Children’s Research Institute (MCRI) in Melbourne, AUS as part of this study. The study protocol included MS-QMA as a first line screening test, followed by AmplideX methylation specific (m)PCR and FREE2 methylation analysis using the EpiTYPER system as second- and third-line tests respectively (Fig. 1B). The MS-QMA results for the U.S.A. cohort were compared to reference ranges from 132 male and 242 female control samples collected as part of previous studies^10,12^. Eight proband FM male samples, two FM female samples and one sex chromosome aneuploidy sample from MCRI were used as positive controls. The study was approved by the Royal Children’s Hospital Research Ethics Committee.

Participants whose samples were referred for fragile X testing at Lineagen (UT, U.S.A) provided written consent when their samples were submitted for clinical testing. These samples were exclusively tested for *FMR1* methylation using MS-QMA (testing for the same condition on the initial test request) anonymously as part of quality control procedures at Lineagen (UT, U.S.A); while AmplideX testing on these samples was performed as part of standard diagnostic practice. Informed consent was obtained from all subjects including Australian positive and negative controls, or if subjects were under 18, it was obtained from a parent and/or legal guardian. All study procedures were in accordance with the Declaration of Helsinki and approved by the Royal Children’s Hospital Human Research Ethics Committee (Single Site: HREC 34227A and HREC 33066F; Multi site HREC: HREC/13/RCHM/24).

### Sample processing and CGG sizing methods

For all AUS reference samples from blood, buccal or saliva, first line *FMR1* testing was performed at Victorian Clinical Genetics Services (VCGS) (MCRI, Melbourne AUS) using a PCR assay with precision of +/−1 repeat and limit of detection at 170 CGG repeats in males and 130 CGG repeats in females^20^. Southern blot analysis reflex testing was performed on samples in the PM range, and inconclusive PCR results including either ‘one peak’ females and ‘no peak’ males^21^.

All infants who provided newborn blood spot (NBS) reference samples were from a general population cohort born at John Hunter Hospital in Newcastle. Extra discs were punched from each child’s NBS cards as part of a fragile X feasibility study by the NSW Newborn Screening Programme and Department of Molecular Genetics at the Children’s Hospital, Westmead. Two PCR methodologies were used to determine CGG size: (i) a modified PCR assay using a chimeric CGG-targeted primer^22^; and (ii) a standard PCR-based fragile X assay^23^, performed in parallel. Alleles with <40 repeats were sized by non-denaturing capillary electrophoresis and alleles with ≥40 CGG repeats were sized using denaturing capillary electrophoresis.

For all U.S.A. samples, the 1^st^ line FXS screening was performed by using the commercial AmplideX *FMR1* PCR kit CGG sizing assay, as per manufacturer’s instructions (Asuragen, Austin, Texas, USA)^24^. Briefly, PCR products were denatured at 95 °C for two minutes after being mixed with a ROX 1000 size standard and Hi-Diformamide. These were then run on ABI PRISM 3730 capillary electrophoresis (Life technologies, Foster City, CA) using POP-7 polymer (Life Technologies) with a 50-cm capillary, according to the manufacturer’s instructions. The CGG sizing was determined using Gene Mapper software version 5.0 (Life Technologies, Foster City, CA). All probands with alleles in the PM range were reflexed for confirmatory testing using AmplideX methylation specific (m)PCR (Fig. 1A).

### First-line testing using Methylation Specific Quantitative Melt Analysis (MS-QMA)

All DNA samples prior to MS-QMA and EpiTYPER methylation analysis (total of 150 ng of DNA per sample) were treated with sodium bisulphite using EZ-96 DNA Methylation-Gold™ (Zymo Research, Irvine, CA), with two separate bisulfite conversions per patient sample, as previously described^25^. Ninety-six samples were bisulfite converted at a time (3 controls and 93 unknown samples per plate) and were serially diluted 4 times post-conversion. Each set of four 96 well plates was then transferred into a 384 well format for real-time PCR analysis utilizing MeltDoctor™ high-resolution melt reagents in 10ul reactions, as per manufacturer’s instructions (Life technologies, Foster City, CA).

For real-time PCR, a unique primer set was used that targets specific CpG sites within the FREE2 region that were previously shown to be most significantly associated with cognitive impairment in FM females^26^. The annealing temperature for the thermal cycling protocol was 65 °C for 40 cycles. The ViiA™ 7 Real-Time PCR System (Life technologies, Foster City, CA) was then used to measure the rate of dye incorporation into double stranded DNA in order to quantify DNA concentration of the unknown samples using the relative standard curve method (Fig. 1B). The dynamic linear range (between 0.05–10 ng/μl) was determined from the standard curve using a series of doubling dilutions of a converted DNA standard from a control lymphoblast cell line during each run. To progress to the next stage of the analysis the unknown samples had to be within this dynamic linear range.

The high resolution melt step followed the real-time PCR in close tube format. The products from methylated and unmethylated FREE2 sequence were then separated into single strands in the temperature range of 74 °C and 82 °C. The High Resolution Melt (HRM) software module for ViiA™ 7 System was then used to plot the rate of PCR product separation to single strands at different temperatures with the difference in fluorescence converted to aligned fluorescence units (AFU) at 78 °C. The AFU conversion to the methylation percentage, and all of the above quality control steps, were analysed simultaneously for 384 reactions at a time using Q-MAX software (Curve Tomorrow, Melbourne, Australia), developed to automate the process^10^.

This software utilizes a custom-designed computer algorithm to simultaneously perform multiple quality control checks to determine DNA concentrations and quality post bisulfite conversion using raw real-time PCR and high resolution melt data for all dilutions from each bisulfite reaction. The high resolution melt data for those sample dilutions outside the quality control (QC) ranges are discarded from the quantitative methylation analysis by the Q-MAX software, and are not used for the final aggregate methylation ratio calculation, as previously described^9,10^. The high resolution melt profiles discarded from quantitative assessments by Q-MAX, however, are used for visual assessments for presence or absence of abnormal methylation as compared to control melt curves. This may reflect variability in replicate melt curves seen for some cases, as previously described^9,10^, and may explain why the methylation ratio derived by Q-MAX is similar for samples that may have differences in in their melt profiles from visual inspection.

Males were considered to have positive MS-QMA results if they had >2% methylation derived through Q-MAX and/or one of more high resolution melt curve profiles that through visual assessment were different from normal size (NS) controls. Specifically, for samples at <2% methylation or in the cases where all melt profiles did not pass QC for automated analysis, all 4 curves were used for qualitative visual assessment of fluorescence (AFU) being above that of the NS controls at 78 °C (single temperature). It is important to note that manual visual assessment is not quantitative, and could not be used to approximated % methylation, as this is provided by the Q-MAX software^9,10^. Through DNA spiking experiments this visual qualitative assessment showed analytical sensitivity of 1%, while analytical sensitivity for the quantitative assessment by the Q-MAX software had analytical sensitivity of 2%^10^. Females were considered positive if they had >37% methylation from quantitative analysis utilizing Q-MAX software.

### Second-line testing of MS-QMA positive samples

All MS-QMA positive DNA samples were re-tested using AmplideX mPCR, (Asuragen, Inc., Austin, TX, U.S.A.), as previously described^27^. Specifically, the AmplideX™ *FMR1* mPCR Kit was used to perform CGG sizing and determine mean methylation at two *HpaII* sites, 5’ and 3’ of the CGG expansion using blood DNA, as per manufacturer’s instructions (Asuragen, Austin, Texas, USA). FREE2 methylation using the EpiTYPER system testing was also performed on the same MS-QMA positive samples, as previously described^4^.

### Microarray analysis

Chromosome microarray analysis (CMA) was performed using FirstStepDx PLUS, a 2.8 million probe ultra-high-resolution microarray optimized for detection of copy number variants (CNVs) related to neurodevelopmental disorders, with analytical and clinical validation previously described^28^. CMA was performed in a CLIA-certified laboratory with DNA extracted by standard methodologies from ORAcollect buccal swab (DNA Genotek, Global). CMA reagents and equipment were as specified by Affymetrix. Microarrays were interpreted by clinical cytogeneticists with established cytogenetic criteria for interpretation as previously described^29^.

### Data analysis

DNA from positive (22 males and 8 females for Lineagen, USA) and negative controls (88 typically developing females and 23 males (FSIQ > 70), from MCRI) were used to determine sensitivity and specificity. Sensitivity and specificity were determined as measures of the probability of correctly identifying the presence or absence of a FM allele and abnormal *FMR1* methylation, respectively, for the control cohorts. Abnormal methylation identified by MS-QMA in absence of a FM was confirmed with one or more of the comparator tests. Comparator tests included FREE2 EpiTYPER based methylation analysis, and AmplideX mPCR. The relationships between MS-QMA and AmplideX mPCR were assessed using ordinary simple linear regression analysis, and used robust standard error to take into account of heterogeneity using the commercial software STATA, version 15 (http://www.stata.com), as in^26^.

## Supplementary information

Supplementary Information
