# Supplementary material for: Abnormally Methylated FMR1 in Absence of a Detectable Full Mutation in a U.S.A Patient Cohort Referred for Fragile X Testing
Source: Sci Rep. 2019 Oct 25;9:15315. doi: 10.1038/s41598-019-51618-7 (PMC6814816; doi:10.1038/s41598-019-51618-7)
Supplement: Supplementary file 1 — Supplementary Information [file 41598_2019_51618_MOESM1_ESM.docx]

**Supplementary Information**

**Title:** Abnormally Methylated *FMR1* in Absence of a Detectable Full Mutation in a U.S.A Patient Cohort Referred for Fragile X Testing.
**Short title:** *Detection of Fragile X Syndrome using MS-QMA.*

Charles H. Hensel, PhD^1^, Rena J. Vanzo MS, CGC^1^, Megan M. Martin, MS, CGC^1^; Ling Ling, MD^2^; Solange M. Aliaga, MS, PhD^2^; Minh Bui, PhD^3^, David I. Francis, MSc, FFSc (RCPA)^4^, Hope Twede, BS^1^; Mike H. Field, MBChB, MPhil^5^ ; Jonathon W. Morison, BSc^6^; David J Amor, MBBS, PhD^7,8^, David E. Godler, PhD^2,7^*.

**Affiliations:**

^1^ Lineagen, Inc. Salt Lake City, UT, United States of America.

^2^ Diagnosis and Development, Murdoch Children’s Research Institute, Royal Children's Hospital, Melbourne, VIC, Australia.

^3^Centre for Epidemiology and Biostatistics, Melbourne School of ^3^Population and Global Health, University of Melbourne, Melbourne, VIC, Australia.

^4^Victorian Clinical Genetics Services and Murdoch Children’s Research Institute, Royal Children's Hospital, Melbourne, VIC, Australia.

^5^Genetics of Learning Disability Service (GOLD service), Hunter Genetics, Newcastle, NSW, Australia.

^6^ Business Development and Legal Office, Murdoch Children’s Research Institute, Royal Children's Hospital.

^7^Faculty of Medicine, Dentistry and Health Sciences, Department of Paediatrics, University of Melbourne, Parkville, VIC, Australia.

^8^Developmental Disability and Rehabilitation Research Group, Murdoch Children’s Research Institute, Royal Children's Hospital, Melbourne, VIC, Australia.

*CORRESPONDING AUTHOR:

A.Prof. David E Godler

The Murdoch Children Research Institute

The Royal Children’s Hospital

50 Flemington Rd, Parkville, Victoria 3052 AUS

Telephone: +61 03 8341 6391

Email: david.godler@mcri.edu.au

**Fig. S1. Raw profiles, CGG sizes and methylation levels for thirteen cases with developmental delay identified to have abnormal methylation using AmplideX mPCR commercial assay.** Upper panels with blue traces representing control digestion [where numbers next to each peak indicate CGG size] and lower panels with green traces representing peaks from *HpaII* digestion reactions [with % next to each peak representing % methylation]. These blue and green traces were used to perform CGG sizing and determine average methylation % across two *HpaII* sites, 5’ and 3’ of the CGG expansion using Gene Mapper software version 5.0 (Life Technologies, Foster City, CA), as per manufacturer’s instructions (Asuragen, Austin, Texas, USA). Note: Pink background represent peaks for alleles of FM CGG size and above (>199 CGGs). Grey background represents peaks for alleles of <200 CGGs.

**Participant ID: P4-B7**

**Participant ID: P3-D7**

**Participant ID: P3-D7 (higher magnification)**

**Participant ID: P2-A11**

**Participant ID: P2-A6**

**Participant ID: P4-E5**

**Participant ID: P4-B8**

**Participant ID: P4-B8 (magnified)**


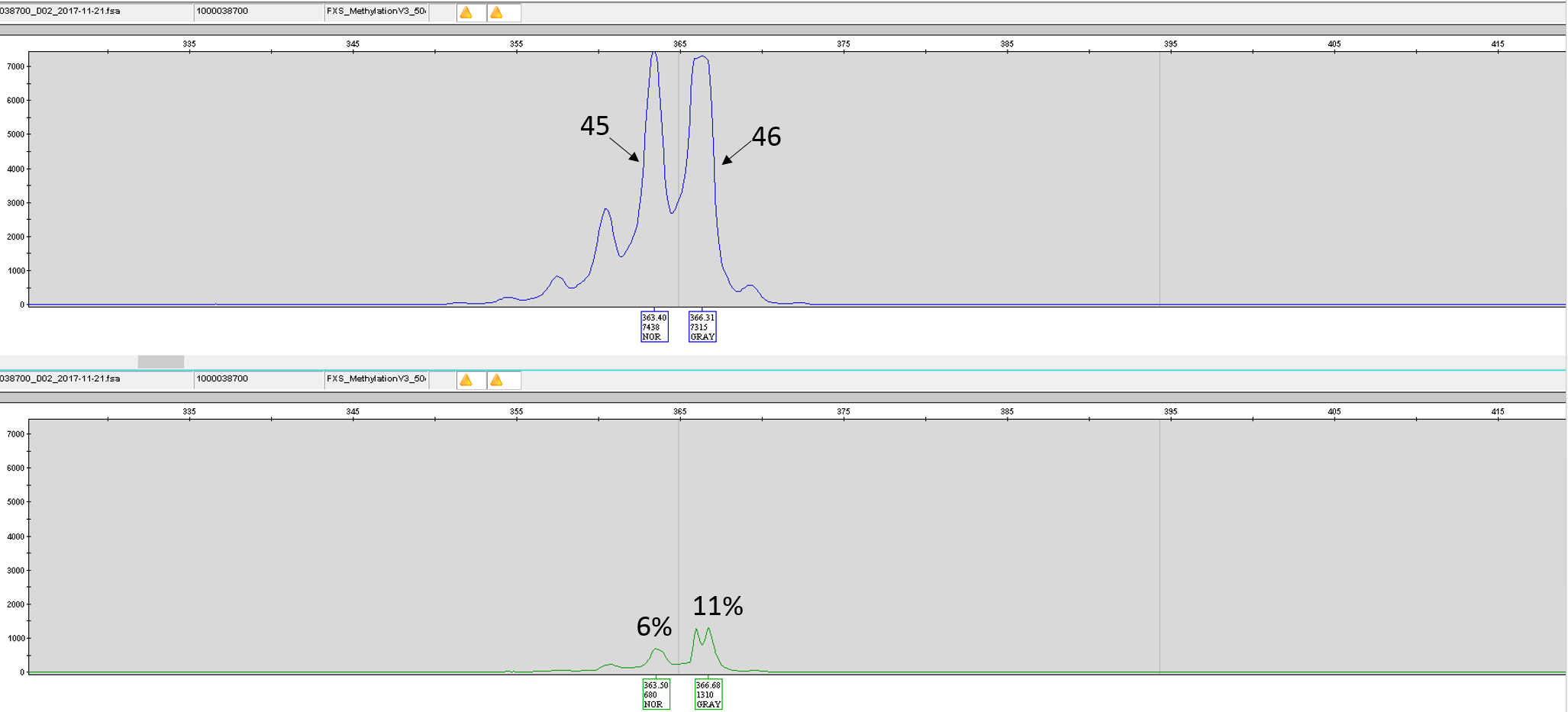


**Participant ID: P1-F12**

**Participant ID: P1-F12 (magnified)**


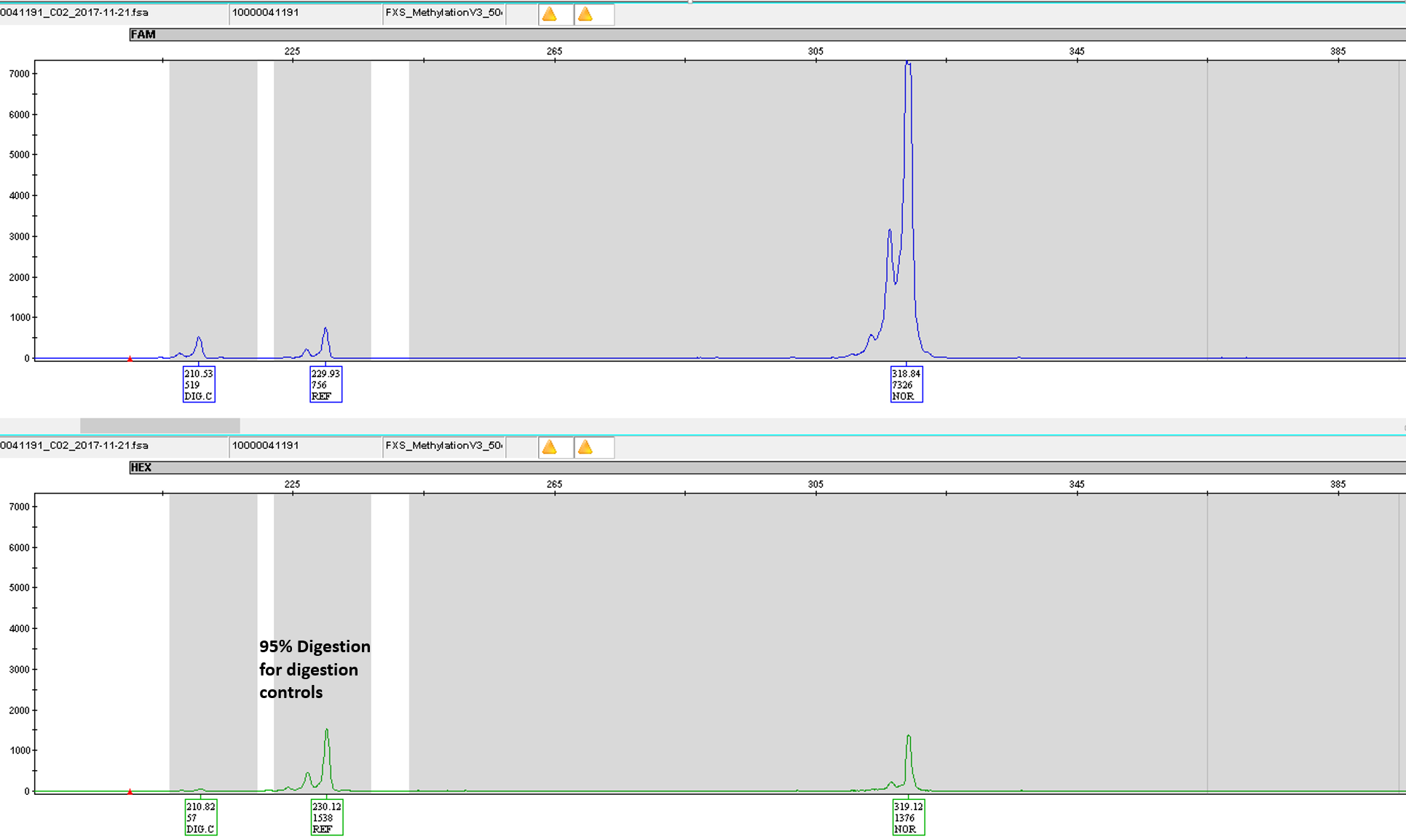


**Participant ID: P4-D4**


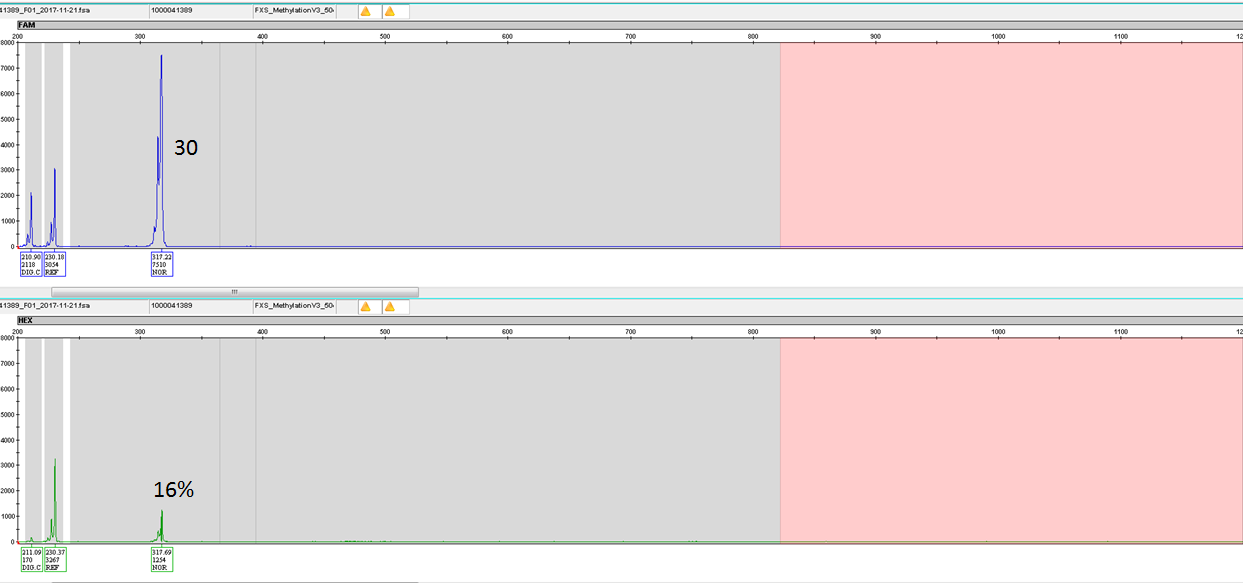


**Participant ID: P1-A11**

**Participant ID: P4-B5**

**Participant ID: P4-D9**

**Participant ID: P4-C6**

**Participant ID: P1-E3**
